# Determinants of Healthcare Professionals’ Intentions to Use Telemedicine for Elderly Care in Algeria: A Technology Acceptance Model Approach

**DOI:** 10.3390/geriatrics11040076

**Published:** 2026-06-27

**Authors:** Kamel Mouloudj, Anuli Njoku, Ahmed Chemseddine Bouarar, Dachel Martínez Asanza, Marian A. Evans, Snehal Baviskar

**Affiliations:** 1 Department of Commercial Sciences, College of Economic, University of Medea, Medea 26000, Algeria; kmouloudj@yahoo.fr (K.M.); bouarar.chemeseddine@univ-medea.dz (A.C.B.); 2 Department of Public Health, College of Health and Human Services, Southern Connecticut State University, New Haven, CT 06515, USA; evansm7@southernct.edu (M.A.E.); gholapsnehal1996@gmail.com (S.B.); 3 Department of Scientific-Technical Results Management, National School of Public Health (ENSAP), Havana Medical Sciences University, Havana 10800, Cuba; dachelmtnez@infomed.sld.cu

**Keywords:** telemedicine, healthcare professionals, elderly care, Technology Acceptance Model (TAM), cross-sectional study, self-efficacy, institutional support, digital health, Algeria

## Abstract

**Background/Objectives**: Telemedicine offers significant potential to improve the quality and accessibility of geriatric care, particularly in resource-constrained settings. However, its effective implementation depends largely on healthcare professionals’ acceptance and willingness to use such systems. Drawing on an extended Technology Acceptance Model (TAM), this study examines the determinants of doctors’ and nurses’ intentions to adopt telemedicine for elderly care in Algeria, with particular emphasis on self-efficacy and institutional support. **Methods**: This cross-sectional study employed a structured questionnaire administered to 130 healthcare professionals, including physicians and nurses, in Algeria. Hierarchical multiple regression analysis was conducted to test the proposed hypotheses and assess the incremental explanatory power of the extended model. **Results**: The extended TAM accounted for 48.7% of the variance in intention to use telemedicine. Institutional support (β = 0.432, *p* < 0.001) and self-efficacy (β = 0.264, *p* = 0.001) emerged as the strongest predictors. Perceived ease of use (β = 0.178, *p* = 0.038) and perceived usefulness (β = 0.139, *p* = 0.021) also had significant positive effects. The inclusion of self-efficacy and institutional support increased the model’s explanatory power by 23.5%. **Conclusions**: The findings highlight the critical role of organizational support mechanisms, digital competencies, and system usability in fostering telemedicine adoption among healthcare professionals. The study provides practical implications for policymakers and healthcare institutions, emphasizing the need for targeted training programs, supportive infrastructure, and institutional policies that enhance confidence and facilitate the integration of telemedicine into clinical workflows.

## 1. Introduction

The increasing use of digital health solutions has created significant opportunities to improve elderly care, particularly through telemedicine [1]. Digital healthcare technologies—including telemedicine—provide several benefits, such as improving patient access and disease management, reducing travel and in-person consultations, lowering costs, and enabling faster response times [2,3,4,5]. Telemedicine allows healthcare practitioners to deliver patient-centered services across long distances using digital technologies, thereby reaching underserved areas and vulnerable population groups such as the elderly [6,7,8]. These systems enable remote monitoring, diagnosis, and treatment through communication technology tools [9,10]. Healthcare professionals who use telemedicine technologies often highlight its “feasibility,” “flexibility,” “cost-effectiveness,” ease of use, and “improved accessibility,” making it a suitable approach for delivering healthcare services [11,12].

However, the successful adoption of telemedicine ultimately depends on healthcare professionals’ willingness to integrate these technologies into their daily practice [13,14]. Rouidi et al. [15], in their systematic review, noted that despite the recognized benefits of telemedicine, low acceptance and resistance among healthcare professionals continue to hinder its widespread use. Similarly, Mohammed et al. [16] emphasized that practitioners’ acceptance of telemedicine is a critical factor for its “successful implementation”.

In Algeria, the Ministry of Health has made substantial efforts to digitize the health sector by investing in equipment, technology, training, research, and institutional support for public hospitals. These national initiatives aim to improve healthcare quality and enhance patient satisfaction in both urban and underserved regions. Nevertheless, Algeria’s vast geographical area (2.38 million km^2^), population growth (approximately 1.39% annually), recent health crises such as COVID-19, and infrastructural limitations in remote regions continue to challenge the provision of equitable healthcare services—particularly for elderly individuals and patients with chronic conditions.

During the COVID-19 pandemic, telemedicine played an essential role in maintaining continuity of care [8,9], particularly for older adults who faced heightened vulnerability to severe illness [17]. Recent studies show that digital platforms not only supported remote monitoring, psychological care, and chronic disease management, but also served as essential tools for assessing the psycho-physical impact of social distancing measures through online data collection and evaluation mechanisms [10,11,12,13,14,18]. At the same time, evidence suggests that maintaining social support through online and offline channels helped preserve individuals’ psychological well-being by sustaining basic psychological needs and reducing anxiety during periods of confinement [18]. Moreover, the use of online platforms enabled the identification of increased levels of anxiety, depression, and reduced quality of life during lockdowns, highlighting the critical need for targeted psychosocial interventions [19]. Importantly, these platforms also mitigated the negative effects of social distancing—such as reduced social support and increased isolation—by enabling virtual check-ins and communication [17,18,19]. These developments highlighted telemedicine’s value not only as a clinical tool but also as a socio-assistive mechanism supporting older adults with limited mobility [11].

A growing number of studies have examined the antecedents of digital health technology adoption among healthcare professionals across various roles (e.g., physicians, nurses, and administrators) [15,20,21]. However, most of this research has been conducted in developed countries [2,22]. Given the economic, cultural, and organizational differences between developed and developing contexts, further investigation is required to understand the determinants influencing healthcare professionals’ adoption of telemedicine solutions [11], particularly for elderly care in countries such as Algeria.

In digital health settings, various theoretical frameworks have been used to assess healthcare practitioners’ perceptions and readiness to adopt new technologies, including the “Unified Theory of Acceptance and Use of Technology” (UTAUT) [2,3,13,16], the “Technology Readiness and Acceptance Model” (TRAM) [23], and the “Theory of Planned Behavior” (TPB) [24]. Additionally, many researchers have applied the “Technology Acceptance Model” (TAM) developed by Davis [25], often extending it with constructs such as accessibility, facilitating conditions, perceived incentives, and subjective norms to enhance its predictive capability [10,11,26]. Systematic reviews by Harst et al. [7] and Rouidi et al. [15] confirm that TAM remains the most widely used framework for examining intentions to use telemedicine.

In line with this context, the primary objective of the present study was to examine the core TAM determinants—“perceived usefulness” (PU) and “perceived ease of use” (PEOU)—that influence healthcare professionals’ intention to use telemedicine for elderly care in Algeria. The secondary objective was to assess the additional contribution of self-efficacy and institutional support, two contextual variables that may enhance the explanatory power of TAM in healthcare settings. Accordingly, the study addressed the following research questions: (1) to what extent do PU and PEOU predict healthcare professionals’ intention to use telemedicine? and (2) how do self-efficacy and institutional support further influence their intention to adopt telemedicine systems for elderly care?

## 2. Literature Review

### 2.1. Using Telemedicine for Elderly Care

With the increasing aging rates in various countries of the world and the lack of healthcare facilities, it will be necessary to consider the well-being of the elderly and improve their quality of life to enable them to live well [27,28,29]. The COVID-19 health crisis has increased interest in implementing technology solutions [30], including telemedicine and virtual care [11,19,31]. Telemedicine can be an effective strategic approach to reducing health disparities in developing societies—provided it is “properly designed”, “developed”, “implemented”, and adapted to the organizational context of these environments [16]. Telemedicine systems can offer a range of services for elderly care, including medical consultations, responding to inquiries, scheduling appointments, providing preventive education, prescribing medications for minor conditions, interpreting X-rays and lab tests, and monitoring patients.

In this context, implementing a telemedicine system offers numerous benefits for both the elderly and healthcare organizations. For example, the benefits for the elderly include easier appointment scheduling, enhanced self-health management, reduced travel requirements and associated costs, improved access to health education, and better quality of interactions with physicians [1,6,11,31]. Meanwhile, healthcare organizations benefit from adopting digital technology in general—and telemedicine in particular—in several ways, including providing timely care, reducing costs, lessening pressure on hospitals, training health staff, and improving operational efficiency [3,10,12,16,24]. Mohan et al. [4] suggest that integrating “deep learning-based cooperative systems” may improve well-being, help assess some risks, and enhance preventive care among the elderly.

On the other hand, “older adults often face challenges and barriers due to physical impairments (poor vision or lower motor control), cognitive difficulties (memory and speed of processing), and a lack of technological skills” ([31], p. 2). Many also experience chronic illnesses, poverty, a lack of self-efficacy, support, and awareness or knowledge [32,33]. Previous investigations have identified low interest in, or barriers to, the use of technology in general—and digital health technology in particular—among the elderly [29]. Aslan et al. [34] concluded that several barriers may hinder older adults from using digital health technology, including a lack of support, limited awareness, low digital literacy, concerns about security and convenience, and a preference for in-person interactions. Furthermore, Almokdad et al. [35] indicated that dehumanized services may generate negative feelings and negatively affect the customer experience, particularly among the elderly elderly. So, the use of telemedicine for geriatric care by healthcare professionals may face several challenges, including usability issues [1], “a lack of standardization”, “human and technological errors”, and insufficient “government policies to support” older adults—particularly in underserved areas [36]. Additionally, Lee et al. [37] identified “lack of trust” and “inadequate training” as key challenges to the adoption of digital health technologies. Healthcare professionals themselves may also lack the necessary skills to effectively use telemedicine [12,38,39].

In fact, using telemedicine may be challenging for older adults—especially those with disabilities, the very elderly, or those living alone. However, unlike many countries such as those in Europe and Japan, where older adults often live independently [4,28], in Algeria and most Arab countries, religious and cultural norms encourage older adults to live with family members. This familial support may help reduce the impact of many cognitive and technological barriers, as support networks—such as family members—can assist with technology use [34]. This cultural factor may positively influence the adoption of telemedicine in Algeria, where family involvement is common.

Beyond accessibility, telemedicine plays a critical role in improving clinical quality outcomes for elderly patients. Research has shown that remote monitoring and virtual consultations can support early detection of complications, enhance treatment adherence, and reduce avoidable hospitalizations through timely follow-up [8]. Telemedicine also enables multidisciplinary collaboration, allowing physicians, nurses, pharmacists, and allied health professionals to coordinate care plans for chronic conditions such as hypertension, diabetes, dementia, and cardiovascular disease—conditions highly prevalent among older adults [14]. Despite these benefits, patient acceptance remains mixed, especially among elderly users who may be hesitant due to limited digital literacy, concerns about diagnostic accuracy, or a preference for in-person interactions [17,18,19]. Although COVID-19 accelerated telemedicine adoption globally, it did not fully resolve these concerns, and public perception in many settings, including developing countries, remains uncertain [8]. These dynamics highlight the need to consider both clinical performance and user acceptance when evaluating telemedicine readiness [9].

### 2.2. Technology Acceptance Model

Technology acceptance refers to individuals’ willingness to voluntarily adopt and use a given technology [40]. TAM theory emphasizes that PEOU and PU are important drivers of user intention to accept technologies [25,41]. TAM helps explain why people accept or reject new technology by focusing on how easy and useful they believe the technology is [40]. Behavioral intention was defined as “the degree to which an individual has formulated conscious plans to perform or not perform some specified behavior in future” [25]. Behavioral intention, in our study, refers to healthcare professionals’ willingness and readiness to adopt and consistently use telemedicine technologies in the delivery of care to elderly patients. Previous studies indicate that positive intentions to use technology positively influence its actual use [5,42]. Healthcare professionals are key stakeholders in telemedicine, and their active involvement in its implementation is essential to ensuring its sustainability and, ultimately, its success [16]. This means that forming positive intentions among healthcare professionals toward telemedicine will increase their likelihood of using telemedicine to provide elderly care services.

Several researchers have extended the TAM by incorporating additional constructs [29]. Lee et al. [37] confirmed that the two core constructs of the TAM—PEOU and PU—were not sufficient to fully explain the willingness to accept digital health technologies. As a result, the expanded TAM used in Bîlbîie et al.’s [11] study accounted for 84.6% of the variance in physicians’ intentions to use telemedicine. In this context, self-efficacy is a key factor that has been consistently integrated into the TAM to examine intentions to adopt new technologies [43,44]. Furthermore, institutional support has been identified as a significant determinant of technology acceptance [23,45,46]. Accordingly, this study extends the TAM by incorporating self-efficacy and institutional support to explore healthcare professionals’ intentions to use telemedicine in the context of elderly care.

### 2.3. Hypothesis Development

#### 2.3.1. Perceived Ease of Use (PEOU)

Davis [25] (p. 320) described PEOU as “the degree to which a person believes that using a particular system would be free of effort”. In our study, PEOU refers to the extent to which healthcare professionals believe that telemedicine systems for elderly care are user-friendly, require minimal effort to learn and operate, and integrate smoothly into their daily workflow. Several previous investigations have revealed that PEOU is a significant determinant of digital health technology acceptance among healthcare workers [11,21,26,43]. However, Sawrikar and Mote [47] found that PEOU does not significantly influence the willingness to use digital health interventions. Accordingly, based on the TAM, we proposed the following hypotheses:

**H1.**  *PEOU has a positive impact on intention to adopt telemedicine in geriatric care.*

#### 2.3.2. Perceived Usefulness (PU)

Davis [25] (p. 320) described PU as “the degree to which a person believes that using a particular system would enhance his or her job performance”. In the context of our study, PU refers to healthcare professionals’ belief that using telemedicine systems for elderly care will enhance their efficiency, improve the quality of patient monitoring, and facilitate timely medical interventions. Mohammed et al. [16] reported that a high level of PU is critical for the adoption of telemedicine by physicians and nurses. Pejić Bach et al. [20] found that perceived time-saving contributes to the adoption of HIS (“hospital information systems”) among healthcare professionals. In addition, several empirical investigations have concluded that PU significantly predicts willingness to accept technology in healthcare settings [10,11,21,26,43,48]. Accordingly, the following hypothesis is formulated:

**H2.**  *PU has a positive impact on intention to adopt telemedicine in geriatric care.*

#### 2.3.3. Self-Efficacy

Self-efficacy was defined as “one’s belief in their ability to effectively use a system” ([11], p. 5). Self-efficacy, in our study, refers to healthcare professionals’ confidence in their ability to effectively use telemedicine technologies for elderly patient care, including remote consultations, digital monitoring, and communication tools. Several investigations have indicated a significant correlation between self-efficacy and behavioral readiness to use digital health technologies [43,44,49,50]. Pikkemaat et al. [24] indicated that enhancing doctors’ self-efficacy positively contributes to their willingness to use telemedicine. In addition, Bîlbîie et al. [11] found that self-efficacy positively predicted PEOU. Rho et al. [26] concluded that self-efficacy predicts physicians’ willingness to use telemedicine through PEOU and PU. Interestingly, Cobb et al. [51] found that self-efficacy had no significant effect on telehealth use and emphasized that efforts to encourage telehealth adoption should focus on removing structural barriers rather than solely attempting to change user attitudes. Hence, we develop the following hypothesis:

**H3.**  *Self-efficacy has a positive impact on intention to adopt telemedicine in geriatric care.*

#### 2.3.4. Institutional Support

Institutional support is defined as “the extent to which employees believe that their organization helps them use a particular system” ([45], p. 1553). In the context of our study, institutional support refers to the availability of technical infrastructure, training opportunities, administrative encouragement, and resource allocation provided by healthcare organizations to facilitate the adoption and use of telemedicine for elderly care. Hsia et al. [52] indicated that institutional support for digital health systems is closely linked to an organization’s understanding of the system’s benefits, which in turn enables the allocation of necessary resources for its implementation. Alsahli and Hor [2] reported that organizational support and infrastructure significantly influence doctors’ willingness to accept the use of digital health technologies. Similarly, Mohammed et al. [16] reported that physicians and nurses are more likely to accept telemedicine when they perceive adequate technical and institutional support is available. Lee et al. [45] demonstrated that perceived organizational support positively influences employees’ acceptance of technology and moderates the relationship between PU and behavioral intentions. In addition, Zhao et al. [46] found that institutional support significantly influences self-efficacy, innovative behavior, and intentions to use technology. Alsahli et al. [13] found that the level of institutional support, including “technical assistance and training”, contributed to shaping positive attitudes and intentions towards the use of digital technology among physicians. Institutional support may serve as an antecedent to both PEOU and PU [41]. Khashan et al. [23] found that perceived organizational support influences doctors’ intentions to use technology through PEOU. Gabbiadini et al. [53] found that organizational support is positively related to PEOU and negatively related to technostress; however, its effect on the intention to use technology was found to be insignificant. In their review, Lee et al. [37] found that a lack of organizational support is one of the key barriers to the adoption of digital health technology. Therefore, we develop the following hypothesis:

**H4.**  *Institutional support has a positive impact on intention to adopt telemedicine in geriatric care.*

## 3. Materials and Methods

This study followed the STROBE guidelines for cross-sectional research, as recommended by the EQUATOR Network [54]. The STROBE checklist was used to guide the transparent reporting of the study design, sampling procedures, measurement instruments, and analytical methods. A completed STROBE checklist is provided as a Supplementary File (see Table S1).

### 3.1. Research Instruments

It is important to note that the present study adopts an administrative and behavioral research design, consistent with the TAM, and therefore does not involve clinical designs. Behavioral intention, as used in TAM, represents a well-established construct for predicting individuals’ willingness to engage in technology-related behavior. The study employed a cross-sectional survey design consistent with STROBE recommendations for observational research [54]. The measurement tool was based on the TAM and some empirical studies in the field of digital healthcare technology adoption. The questionnaire included an introduction that covered the purposes of this investigation, emphasized the confidentiality of respondents’ information, and informed them of the possibility of withdrawing at any time before completing the answers. The questionnaire was divided into two sections. The first section was devoted to collecting general personal data such as gender, age, occupation (doctor or nurse), and professional experience in the healthcare field. The second section measured the study constructs, where PEOU was measured by adapting a scale derived from Davis [25] and Bîlbîie et al. [11]. PU was measured using a scale adapted from Davis [25] and Rho et al. [26]. Furthermore, we used a modified scale from Bouarar et al. [43] to measure self-efficacy. Institutional support was measured using a scale derived from Lee et al. [45] and Zhao et al. [46]. Finally, behavioral intentions were measured with the scale drawn from Bîlbîie et al. [11]. All items were measured on a five-point Likert scale, “(1) strongly disagree and (5) strongly agree”, with three items. Measurement procedures and reliability assessments were described following STROBE specifications for clarity and reproducibility.

After designing the measurement tool, two digital health experts were invited to provide suggestions for improving its content, and accordingly, some language and style corrections were made. The questionnaire was developed in English, and since the respondents’ native language was Arabic, we back-translated the questionnaire items to ensure the accuracy of the translation [35]. Before distributing the questionnaire, we conducted a pilot study with 24 participants (12 doctors and 12 nurses) to verify the clarity and accuracy of the measurement items. Based on the participants’ feedback and comments, we made some minor revisions to the style and content. Table 1 shows the measurement items.

### 3.2. Sample and Procedure

The population of this investigation consists of all doctors and nurses working in a public or private hospital in northern Algeria. Due to the lack of a sampling frame and limited resources, we decided to use a convenience sample method [55], considering representativeness in terms of occupation type, gender, age, and other characteristics. This sampling method was chosen due to the practical constraints of reaching geographically dispersed professionals and is commonly employed in administrative and behavioral studies examining technology acceptance [43]. Eligible participants were healthcare professionals currently involved in patient care and with some level of exposure to digital health or telemedicine tools. Individuals not directly involved in clinical care or who declined participation were excluded. Therefore, participant selection and eligibility criteria were reported in accordance with STROBE guidelines.

With the help of four health workers, the questionnaire was distributed directly to participants in private clinics, public hospitals, health centers, and medical laboratories. Accordingly, we distributed 180 paper copies of the questionnaire during January 2025, receiving 132 copies, with a response rate of 73.33%. Data completeness was assessed during the data-cleaning process. Because the survey was self-administered on paper, questionnaires were manually checked for missing responses or inconsistencies. A total of two copies were excluded because they contained unanswered items or incomplete sections. Only fully completed questionnaires (*n* = 130) were retained for analysis. As a result, no item-level missing data were present, no imputation was necessary, and all statistical analyses were performed on complete cases. The sample size is adequate for the hierarchical multiple regression analysis used in this study. Based on the widely accepted rule of thumb suggesting a minimum of 10–15 participants per predictor variable in regression models, the study’s four predictors (PU, PEOU, self-efficacy, and institutional support) would require approximately 60 participants. Thus, the final sample of 130 respondents comfortably exceeds methodological recommendations and supports the stability and reliability of the statistical estimates. Completing the questionnaire took only ten minutes. However, respondents were allowed to complete it in their free time to avoid random responses that may occur due to lack of concentration and/or work pressure.

### 3.3. Ethical Considerations

This study adhered to the ethical principles of confidentiality, voluntary participation, and informed consent. In line with local laws and regulations, “ethical approval was not required” because we used a questionnaire as a data collection tool, ensured that participants’ identities were anonymous, and our study “does not involve clinical trials or interventions” [43]. All participants were informed of the study objectives, the anonymous and confidential nature of their responses, and their right to decline or withdraw at any point without consequences. Verbal consent was obtained from all respondents before completing the paper-based questionnaire. No personal identifiers were collected, and all completed questionnaires were stored securely and accessed only by the research team. All procedures complied with national ethical standards for research involving human participants.

### 3.4. Statistical Analysis

Statistical analyses were conducted using SPSS version 26.0 (“IBM Corporation, Armonk, NY, USA”). Data were analyzed using descriptive statistics, including frequencies, percentages, means, and standard deviations (SD). Reliability was assessed using Cronbach’s alpha. Skewness and kurtosis statistics were used to evaluate normality. The “variance inflation factor” (VIF) was employed to detect multicollinearity. Similarly to the study by Sawrikar and Mote [47], “hierarchical regression analyses” (HRA) were conducted to test the hypotheses. A *p*-value of less than 0.05 was considered statistically significant.

## 4. Results

### 4.1. Demographic Characteristics

Table 2 presents the demographic characteristics of the 130 respondents who participated in the study. The gender distribution shows a slight predominance of female participants (56.15%) over males (43.85%). In terms of age, the majority of respondents (45.38%) were between 31 and 45 years old, followed by those aged 46 to 60 (33.85%), and the youngest group aged 18 to 30 (20.77%). Regarding occupational roles, the sample was almost evenly split, with doctors comprising 51.54% and nurses 48.46% of the participants. In terms of professional experience, 31.54% of respondents had 11 to 15 years of experience, while 28.46% had over 15 years. Additionally, 27.69% had between 5 and 10 years of experience, and a smaller proportion (12.31%) had less than 5 years.

### 4.2. Descriptive Analysis

Table 3 presents the descriptive statistics, reliability coefficients, and correlation matrix for the study variables. All constructs demonstrated acceptable internal consistency, with Cronbach’s alpha values exceeding the recommended threshold of 0.70 [55], indicating reliable measurement. The skewness and kurtosis values for all constructs fall within the acceptable ranges (“±2 for skewness and ±7 for kurtosis”), suggesting that the data are normally distributed [55].

Focusing on the relationships between antecedents and behavioral intention, the strongest correlation was found between institutional support and intention (*r* = 0.636, *p* < 0.01), highlighting the critical role of organizational factors in encouraging telemedicine adoption. Self-efficacy also showed a strong and significant association with intention (*r* = 0.479, *p* < 0.01). PEOU demonstrated a moderate and significant correlation with intention (*r* = 0.469, *p* < 0.01). In contrast, PU exhibited a weaker but still significant correlation with intention (*r* = 0.268, *p* < 0.01). Notably, the relationship between PEOU and PU was relatively weak (*r* = 0.130, not significant). This finding suggests that, in the context of elderly care, PEOU may directly influence intention, rather than exerting its effect via PU.

### 4.3. Testing Hypotheses

Table 4 presents the results of the HRA conducted to examine the effects of key antecedents on healthcare professionals’ intentions to use telemedicine for elderly care. In Model 1, PEOU and PU were both significant predictors of intention, with PEOU (β = 0.506, *p* < 0.001) showing a stronger influence than PU (β = 0.196, *p* = 0.007). This model explained 25.2% of the variance in intention (R^2^ = 0.252), indicating a moderate explanatory power when only TAM constructs are considered.

In Model 2, self-efficacy and institutional support were added to the model, resulting in a substantial increase in explained variance (R^2^ = 0.487). All four predictors remained significant, with institutional support (β = 0.432, *p* < 0.001) emerging as the strongest predictor, followed by self-efficacy (β = 0.264, *p* = 0.001). While the effects of PEOU (β = 0.178, *p* = 0.038) and PU (β = 0.139, *p* = 0.021) were slightly reduced, they remained significant; therefore, H1, H2, H3 and H4 are supported.

Multicollinearity was not a concern in either model, as all VIF values were well below the threshold of 5, and all tolerance values exceeded the minimum acceptable level of 0.2, as recommended by Hair et al. [56]. These diagnostics confirm the independence of the predictors and support the robustness of the regression results.

## 5. Discussion, Implications and Limitations

### 5.1. Discussion

This study examined the determinants of healthcare professionals’ intentions to use telemedicine for elderly care by applying an extended TAM. Consistent with the study’s primary objective, both PU and PEOU significantly predicted intention to adopt telemedicine. Regarding the secondary objective, the addition of self-efficacy and institutional support substantially strengthened the model’s explanatory power, with institutional support emerging as the strongest predictor.

Consistent with TAM theory, our findings support the positive effect of PEOU on healthcare professionals’ behavioral intentions to use telemedicine. Therefore, it appears that the successful adoption of digital technology, including telemedicine technology for elderly care, depends on healthcare providers being able to use modern technology easily [22,36,48]. Furthermore, it will be necessary to familiarize this segment (i.e., the elderly) with the use of technological healthcare tools to build familiarity and become accustomed to their use. This finding supports the findings of several investigations in the context of digital healthcare [10,11,21,26,29,43], which have demonstrated that technology use is influenced by its ease of use [46,57,58].

Moreover, in line with the TAM, our findings confirm the positive impact of PU on behavioral intentions to use telemedicine among healthcare professionals. Our results support the findings of several previous studies, which reported that PU is a significant antecedent of behavioral intentions to adopt technology [8,10,11,21,26,43,47,48]. For healthcare professionals, the PU of telemedicine relate to its effectiveness in simplifying diagnosis, monitoring diseases, and improving treatment outcomes [7,22,57,58]. Therefore, the benefits of using telemedicine for elderly care may include, but are not limited to, improved access to care, enhanced monitoring and early intervention, increased efficiency and time management, better multidisciplinary collaboration, greater patient engagement and satisfaction, reduced exposure to infectious diseases, and improved support for managing chronic conditions such as diabetes, hypertension, and heart disease—common among older adults. In practice, several factors contribute to maximizing the PU of telemedicine use among physicians, including PEOU, subjective norms, self-efficacy, and accessibility [11,26].

While this study focuses on psychological and organizational predictors of telemedicine acceptance, it is also important to consider broader clinical and patient-centered factors influencing its sustainability. Evidence indicates that telemedicine enhances continuity of care, diagnostic quality, and chronic disease management through remote monitoring technologies [8,58]. Furthermore, experiences during the COVID-19 pandemic highlight that digital platforms played a dual role by supporting both clinical services and psychosocial well-being; for example, they enabled the assessment of anxiety, depression, and quality of life during lockdowns while also maintaining social support and reducing psychological distress [18,19]. These findings underscore the value of telemedicine as both a clinical and socio-assistive tool. However, despite these advantages, patient acceptance, particularly among older adults, remains uneven [18,19,48,59], with concerns related to diagnostic reliability, privacy, and the perceived impersonality of remote care [60]. Therefore, promoting telemedicine adoption requires not only strengthening healthcare professionals’ acceptance but also enhancing patient trust, digital literacy, and social connectedness to ensure both clinical effectiveness and psychosocial well-being [14].

Furthermore, empirical analysis indicated that healthcare professionals’ self-efficacy significantly influences their intentions to engage in telemedicine for the care of older adults. This finding supports the results of numerous studies that have confirmed that self-efficacy contributes to positive intentions toward technology use [5,43,44,46,49,50]. Pikkemaat et al. [24] confirmed that doctors’ self-efficacy was the strongest predictor of their intention to use telemedicine. Telemedicine self-efficacy centers on users’ belief in their ability to overcome usage challenges and build self-confidence [51]. In fact, the self-efficacy of various healthcare practitioners to use digital technology, particularly doctors and nurses, can be improved and developed, given their higher education [38]. van Houwelingen et al. [39] found that training enhances self-efficacy for telemedicine among nurses. In contrast, healthcare professionals may face some challenges associated with older adults’ low self-efficacy [31,61]. Physical and mental disabilities can present significant challenges to older adults’ effective use of digital technologies [34], requiring care professionals to exercise patience, invest additional effort, and prioritize the human and emotional aspects of interacting with this population. Therefore, it will be necessary to educate and train older adults to gain confidence in their ability to use technology [22,36,49]. In this context, digital technology can be effectively leveraged to educate, train, and improve self-efficacy among older adults [6,61]. Napetschnig et al. [62] confirmed that effective training within the virtual reality has the potential to improve older adults’ skills and increase their level of self-efficacy, thus improving their overall quality of life.

Finally, our results revealed that institutional support was the strongest predictor of intentions to engage in telemedicine for elderly care. This finding aligns with the results of several previous studies. For instance, Lee et al. [45] and Zhao et al. [46] asserted that employees who perceive strong organizational support are more likely to be willing to adopt new technologies. Almokdad and Lee [30] indicated that organizational support can mitigate the negative impact of perceived burdensomeness. Hsia et al. [52] argue that hospital top management’s conviction in the benefits—both economic and non-economic—of a digital health system can lead them to take bold steps to support its adoption. Mohammed et al. [16] indicated that the availability of organizational and technical support—such as advanced equipment and ongoing training—plays an important role in shaping doctors’ and nurses’ willingness to accept telemedicine. This finding implies that a lack of institutional support may limit healthcare professionals’ willingness to actively engage in telemedicine for elderly care. Based on participants’ responses, the mean score for institutional support was 3.16, indicating a perceived insufficiency of support. This suggests that many healthcare organizations do not provide adequate facilitating conditions or actively encourage the use of telemedicine. Consequently, we argue that institutional support is a critical prerequisite for any initiative aimed at implementing digital health technologies. The absence of such support may stem from fear or a lack of conviction among some healthcare organization leaders.

### 5.2. Practical Implications

The findings of this study provide several evidence-based implications for policymakers, healthcare institutions, and technology developers seeking to promote the effective adoption of telemedicine for elderly care. First, the significant effect of PEOU suggests that telemedicine systems must be designed with usability and simplicity in mind. In fact, user-friendly interfaces and clear system functionalities reduce cognitive effort and enhance technology acceptance among healthcare professionals [58]. Accordingly, healthcare institutions and software developers should prioritize intuitive platform design, clear navigation structures, and high-quality audiovisual functionalities that facilitate remote consultations. Additionally, the provision of continuous technical support, user training, and system maintenance has been shown to reduce resistance to new technologies and improve user experience [26,32,63].

Second, the positive influence of PU highlights the importance of clearly demonstrating the clinical and operational benefits of telemedicine. So, healthcare professionals are more likely to adopt telemedicine when they perceive it as improving diagnostic accuracy, patient monitoring, and treatment outcomes. Institutions should therefore promote evidence-based use cases, particularly in managing chronic conditions prevalent among older adults (e.g., diabetes, hypertension, and cardiovascular diseases), and disseminate success stories and best practices that illustrate improvements in efficiency, access to care, and patient outcomes.

Third, self-efficacy emerged as a strong predictor of telemedicine adoption, emphasizing the need to enhance healthcare professionals’ confidence in using digital health technologies. To address this, healthcare organizations should implement structured training programs, including “simulation-based training” (SBT), hands-on workshops, and guided system demonstrations. Peer learning and mentoring initiatives can further reinforce skills acquisition, reduce technology-related anxiety, and foster positive attitudes toward telemedicine use.

Fourth, institutional support was identified as the strongest determinant of behavioral intention, underscoring the critical role of organizational readiness in technology adoption. Policymakers and hospital administrators should therefore invest in digital infrastructure, ensure the availability of necessary equipment, and establish clear policies and guidelines that support telemedicine integration into clinical workflows [58,63]. Furthermore, targeted training programs addressing the specific needs of elderly patients, such as sensory, cognitive, and mobility limitations, can enhance both perceived ease of use and effectiveness.

Finally, the broader lessons from the COVID-19 pandemic further reinforce the importance of integrating telemedicine within a holistic and patient-centered care model. Therefore, telemedicine initiatives should be embedded within multidisciplinary care frameworks that involve physicians, nurses, psychologists, and social workers, enabling coordinated care for elderly patients with complex needs. Such integration enhances continuity of care, strengthens patient engagement, and ensures that telemedicine functions as both a clinical and socio-assistive tool.

In general, our findings suggest that successful telemedicine implementation requires a comprehensive strategy that combines technological design, capacity building, and strong institutional support, while also addressing the clinical and psychosocial needs of elderly patients.

### 5.3. Limitations and Future Research

Despite its significance, this study has several limitations that open avenues for further investigation. First, the sample was obtained through a convenience approach and included healthcare professionals from selected institutions, which may limit the generalizability of the results to all healthcare settings in Algeria. Future studies using probabilistic or multi-regional sampling strategies would help strengthen external validity. Second, the study relies on self-reported measures, which may be subject to recall bias or social desirability bias. Although anonymity was ensured, responses may still reflect participants’ perceptions more than their actual behaviors. Third, the cross-sectional design does not allow for causal inferences regarding the relationships between TAM variables and the intention to use telemedicine. Mixed-method designs could provide more comprehensive evidence. In addition, while our study focused on the drivers of acceptance, future research should also explore the determinants of resistance to telemedicine, in order to gain deeper insight into potential barriers. Fourth, although this study examined telemedicine acceptance from the perspective of healthcare professionals—who are indeed critical to successful implementation—the capabilities and acceptance of older adults are equally essential [27]. Future research should therefore also examine the factors influencing telemedicine or AI-enabled medicineadoption among elderly users.

Finally, the study focuses on administrative and behavioral determinants of telemedicine adoption rather than clinical outcomes. While this focus aligns with the study’s objectives, it means that interpretations cannot be extended to clinical effectiveness or patient-level results. Despite these limitations, the study provides important initial insights into the organizational and professional factors that influence telemedicine adoption in elderly care contexts.

## 6. Conclusions

This study examined the behavioral and organizational determinants influencing healthcare professionals’ intention to adopt telemedicine for elderly care in Algeria using an extended TAM. The findings underscore the significant roles of institutional support and self-efficacy, alongside PU and PEOU, in shaping professionals’ readiness to integrate telemedicine into clinical practice. These results highlight that successful implementation depends not only on system functionality but also on organizational support mechanisms and users’ confidence in their digital competencies. In the context of elderly care, telemedicine offers important benefits, including improved continuity of care, enhanced coordination among multidisciplinary teams, and better support for patients with chronic conditions or limited mobility. Evidence from the COVID-19 pandemic further demonstrated the value of digital platforms in maintaining follow-up care, facilitating communication, and supporting patients’ psychosocial well-being. From a practical perspective, strengthening telemedicine adoption requires targeted investments in training, digital literacy, and supportive institutional frameworks. Such efforts can enhance healthcare professionals’ confidence and foster more effective integration of telemedicine into routine workflows.

Overall, this study contributes to the growing literature by emphasizing the combined importance of behavioral and organizational factors in telemedicine adoption, offering insights for developing more resilient and patient-centered healthcare systems for aging populations.

## Figures and Tables

**Table 1:** Measurement items.

Constructs	Items	Sources
Perceived Ease of Use (PEOU)	PEOU1. Learning to use telemedicine for elderly care would be easy for me.	Davis [25], Bîlbîie et al. [11]
	PEOU2. I find telemedicine systems intuitive and user-friendly.	
	PEOU3. My interaction with telemedicine platforms would be clear and understandable.	
Perceived Usefulness (PU)	PU1. Using telemedicine would improve the efficiency of elderly patient care.	Davis [25], Rho et al. [26]
	PU2. Telemedicine would enable me to provide high-quality healthcare services.	
	PU3. Telemedicine would enhance my ability to monitor and manage elderly patients.	
Self-Efficacy (SE)	SE1. I am confident in my ability to use telemedicine technologies for elderly care.	Bouarar et al. [43]
	SE2. I believe I can troubleshoot basic issues that arise while using telemedicine.	
	SE3. I feel capable of integrating telemedicine into my daily professional routine.	
Institutional Support (IS)	IS1. My healthcare institution provides adequate resources for implementing telemedicine.	Lee et al. [45], Zhao et al. [46]
	IS2. I receive sufficient training and technical support to use telemedicine effectively.	
	IS3. My organization encourages and supports the adoption of telemedicine for elderly care.	
Behavioral Intention (BI)	BI1. I intend to use telemedicine for elderly care whenever possible.	Bîlbîie et al. [11]
	BI2. I will recommend telemedicine solutions to my colleagues for geriatric care.	
	BI3. I am willing to integrate telemedicine into my routine healthcare practices for elderly patients.	

**Table 2:** Demographic characteristics (*N* =130).

Variable	Categories	Frequency	%
Gender	Female	73	56.15
	Male	57	43.85
	Total	130	100
Age	18–30 years	27	20.77
	31–45 years	59	45.38
	46–60 years	44	33.85
	Total	130	100
Occupation	Doctors	67	51.54
	Nurses	63	48.46
	Total	130	100
Professional Experience	Less than 5 years	16	12.31
	5–10 years	36	27.69
	11–15 years	41	31.54
	Over 15 Years	37	28.46
	Total	130	100

**Table 3:** Descriptive statistics and correlation matrix.

Constructs	PEOU	PU	SE	IS	BI
PEOU	-				
PU	0.130	-			
SE	0.304 **	0.081	-		
IS	0.513 **	0.183 *	0.409 **	-	
BI	0.469 **	0.268 **	0.479 **	0.636 **	-
Cronbach’s Alphas	0.799	0.807	0.883	0.740	0.880
Mean	3.35	3.64	3.17	3.16	3.14
SD	0.45	0.56	0.48	0.51	0.52
Skewness	0.857	0.383	0.646	1.556	1.107
Kurtosis	0.805	−0.336	0.950	3.753	1.984

Note: ** correlation is significant at the 0.01 level; * correlation is significant at the 0.05 level; PEOU = perceived ease of use; PU = perceived usefulness; SE = self-efficacy; IS = institutional support; BI = behavioral intention; SD = standard deviation.

**Table 4:** Hierarchical regression analysis results.

Models	Constructs	B	t	Sig	Tolerance	VIF	F (Adjusted R^2^)
Model 1	(constant)	0.729	1.976	0.050			F = 22.741 (*p* < 0.001); (R^2^ = 0.252)
	PEOU	0.506	5.750	0.000	0.983	1.017	
	PU	0.196	2.748	0.007	0.983	1.017	
Model 2	(constant)	−0.170	−0.502	0.61			F = 49.311 (*p* < 0.001); (R^2^ = 0.487)
	PEOU	0.178	2.094	0.038	0.725	1.379	
	PU	0.139	2.337	0.021	0.965	1.036	
	SE	0.264	3.503	0.001	0.820	1.219	
	IS	0.432	5.517	0.000	0.655	1.527	

Note: PEOU = “perceived ease of use”; PU = “perceived usefulness”; SE = self-efficacy; IS = institutional support.

## Data Availability

The original contributions presented in this study are included in the article/Supplementary Material. Further inquiries can be directed to the corresponding author.

## Supplementary Materials

The following supporting information can be downloaded at: https://www.mdpi.com/article/10.3390/geriatrics11040076/s1, Table S1: STROBE Checklist.

## References

[1] Alharthi S.A. (2025). mHealth Applications in Saudi Arabia: Current Features and Future Opportunities. Healthcare.

[2] Alsahli S., Hor S.Y. (2024). The adoption of mobile health applications by physicians during the COVID-19 pandemic in developing countries: The case of Saudi Arabia. Int. J. Inf. Manag. Data Insights.

[3] Azam M., Bin Naeem S., Kamel Boulos M.N., Faiola A. (2023). Modelling the Predictors of Mobile Health (mHealth) Adoption among Healthcare Professionals in Low-Resource Environments. Int. J. Environ. Res. Public Health.

[4] Mohan D., Chong P.H.J., Gutierrez J. (2025). A Novel Cooperative AI-Based Fall Risk Prediction Model for Older Adults. Sensors.

[5] Yap Y.-Y., Tan S.-H., Choon S.-W. (2022). Elderly’s Intention to Use Technologies: A Systematic Literature Review. Heliyon.

[6] Bertolazzi A., Quaglia V., Bongelli R. (2024). Barriers and facilitators to health technology adoption by older adults with chronic diseases: An integrative systematic review. BMC Public Health.

[7] Harst L., Lantzsch H., Scheibe M. (2019). Theories predicting end-user acceptance of telemedicine use: Systematic review. J. Med. Internet Res..

[8] Kung L.-H., Yan Y.-H., Kung C.-M. (2024). Exploring Telemedicine Usage Intention Using Technology Acceptance Model and Social Capital Theory. Healthcare.

[9] Angelopoulou E., Kontaxopoulou D., Fragkiadaki S., Stanitsa E., Pavlou D., Papatriantafyllou J., Koros C., Dimovski V., Šemrov D., Papageorgiou S.G. (2024). Perceptions of Patients, Caregivers, and Healthcare Professionals toward Telemedicine Use for Cognitive and Movement Disorders in the Aegean Islands, Greece: A Pilot Study of the SI4CARE European Project. Geriatrics.

[10] He H., Raja Ghazilla R.A., Abdul-Rashid S.H. (2025). Factors influencing the intention to use telemedicine services among older adults in China. Sci. Rep..

[11] Bîlbîie A., Puiu A.-I., Mihăilă V., Burcea M. (2024). Investigating Physicians’ Adoption of Telemedicine in Romania Using Technology Acceptance Model (TAM). Healthcare.

[12] Konidari E., Adrion E., Kontogianni E., Alexaki M., Aggeletaki E., Gkampra M., Delatola M., Delatolas A., Efkarpidis A., Alokrios G. (2025). Viewpoints of Healthcare Professionals on Care Delivery Within the Frames of Old-Age Mental Telehealth Services Operating in Low-Resource Settings. Brain Sci..

[13] Alsahli S., Hor S.Y., Lam M.K. (2024). Physicians’ acceptance and adoption of mobile health applications during the COVID-19 pandemic in Saudi Arabia: Extending the unified theory of acceptance and use of technology model. Health Inf. Manag. J..

[14] Offermann J., Ziefle M., Optimal@NRW Research Group (2024). You are not alone! Care professionals’ acceptance of telemedicine in nursing homes comparing pre- and post-implementation evaluations. Electronics.

[15] Rouidi M., Hamdoune A., Choujtani K., Chati A. (2022). TAM-UTAUT and the acceptance of remote healthcare technologies by healthcare professionals: A systematic review. Inform. Med. Unlocked.

[16] Mohammed R., Elmajid E.A., Amine H., Khadija C. (2023). Acceptance Factors of Telemedicine Technology during COVID-19 Pandemic among Health Professionals: A Qualitative Study. Healthc. Technol. Lett..

[17] Zakrzewski K.M., Mularczyk-Tomczewska P., Koweszko T., Czyżewski Ł., Silczuk A. (2025). Patterns of healthcare use and disease burden among older adults in Poland: A large-scale retrospective study of primary care utilization. Geriatrics.

[18] Costa S., Canale N., Mioni G., Cellini N. (2022). Maintaining social support while social distancing: The longitudinal benefit of basic psychological needs for symptoms of anxiety during the COVID-19 outbreak. J. Appl. Soc. Psychol..

[19] Scuri S., Tesauro M., Petrelli F., Argento N., Damasco G., Cangelosi G., Nguyen C.T.T., Savva D., Grappasonni I. (2022). Use of an online platform to evaluate the impact of social distancing measures on psycho-physical well-being in the COVID-19 era. Int. J. Environ. Res. Public Health.

[20] Pejić Bach M., Mihajlović I., Stanković M., Khawaja S., Qureshi F.H. (2024). Determinants of Intention to Use of Hospital Information Systems among Healthcare Professionals. Systems.

[21] Phorah K.E., Motsi L. (2025). Acceptance and adoption determinants of telemedicine in public healthcare institutions. Curationis.

[22] Bunnell B.E., Barrera J.F., Paige S.R., Turner D., Welch B.M. (2020). Acceptability of Telemedicine Features to Promote Its Uptake in Practice: A Survey of Community Telemental Health Providers. Int. J. Environ. Res. Public Health.

[23] Khashan M.A., Alasker T.H., Ghonim M.A., Elsotouhy M.M. (2025). Understanding physicians’ adoption intentions to use Electronic Health Record (EHR) systems in developing countries: An extended TRAM approach. Mark. Intell. Plan..

[24] Pikkemaat M., Thulesius H., Milos Nymberg V. (2021). Swedish primary care physicians’ intentions to use telemedicine: A survey using a new questionnaire–physician attitudes and intentions to use telemedicine (PAIT). Int. J. Gen. Med..

[25] Davis F.D. (1989). Perceived usefulness, perceived ease of use, and user acceptance of information technology. MIS Q..

[26] Rho M.J., Choi I.Y., Lee J. (2014). Predictive Factors of Telemedicine Service Acceptance and Behavioral Intention of Physicians. Int. J. Med. Inform..

[27] Lu C.C., Tsai-Lin T.F. (2024). Are older adults special in adopting public eHealth service initiatives? The modified model of UTAUT. Sage Open.

[28] Naoe M., Kawahara Y. (2025). Effective Social Support to Enable Older Adults Living Alone in Japan to Continue Living at Home. Int. J. Environ. Res. Public Health.

[29] Wang A., Zhou Y., Ma H., Tang X., Li S., Pei R., Piao M. (2024). Preparing for aging: Understanding middle-aged user acceptance of AI chatbots through the technology acceptance model. Digit. Health.

[30] Almokdad E., Lee C.H. (2024). Service robots in the workplace: Fostering sustainable collaboration by alleviating perceived burdensomeness. Sustainability.

[31] Zin K.S.L.T., Kim S., Kim H.-S., Feyissa I.F. (2023). A Study on Technology Acceptance of Digital Healthcare among Older Korean Adults Using Extended Tam (Extended Technology Acceptance Model). Adm. Sci..

[32] Asanza D.M., Njoku A., Baviskar S., Evans M.A., Mouloudj K. (2025). Oral hygiene care of older adults and caregiver education: A systematic review. Hygiene.

[33] Park C., Park S. (2026). The technological-personal-environmental framework applied to predicting older adults’ telemedicine utilization. Educ. Gerontol..

[34] Aslan A., Mold F., van Marwijk H., Armes J. (2024). What are the determinants of older people adopting communicative e-health services: A meta-ethnography. BMC Health Serv. Res..

[35] Almokdad E., Mouloudj K., Lee C.H. (2025). Rehumanizing AI-driven service: How employee presence shapes consumer perceptions in digital hospitality settings. J. Theor. Appl. Electron. Commer. Res..

[36] Doraiswamy S., Jithesh A., Mamtani R., Abraham A., Cheema S. (2021). Telehealth Use in Geriatrics Care during the COVID-19 Pandemic—A Scoping Review and Evidence Synthesis. Int. J. Environ. Res. Public Health.

[37] Lee A.T., Ramasamy R.K., Subbarao A. (2025). Understanding Psychosocial Barriers to Healthcare Technology Adoption: A Review of TAM Technology Acceptance Model and Unified Theory of Acceptance and Use of Technology and UTAUT Frameworks. Healthcare.

[38] Abildgren L., Lebahn-Hadidi M., Mogensen C.B., Toft P., Nielsen A.B., Frandsen T.F., Steffensen S.V., Hounsgaard L. (2022). The effectiveness of improving healthcare teams’ human factor skills using simulation-based training: A systematic review. Adv. Simul..

[39] van Houwelingen T., Ettema R.G., Bleijenberg N., van Os-Medendorp H., Kort H.S., Ten Cate O. (2021). Educational intervention to increase nurses’ knowledge, self-efficacy and usage of telehealth: A multi-setting pretest-posttest study. Nurse Educ. Pract..

[40] Gelibolu M., Mouloudj K., Bouarar A. (2026). Drivers of Customer Adoption for Food Ordering Apps: A Systematic Literature Review. Insights on Consumer Psychology in the Digital Landscape.

[41] Davis F.D., Bagozzi R.P., Warshaw P.R. (1989). User acceptance of computer technology: A comparison of two theoretical models. Manag. Sci..

[42] Venkatesh V., Morris M.G., Davis G.B., Davis F.D. (2003). User acceptance of information technology: Toward a unified view. MIS Q..

[43] Bouarar A.C., Mouloudj S., Umar T.P., Mouloudj K. (2023). Antecedents of physicians’ intentions to engage in digital volunteering work: An extended technology acceptance model (TAM) approach. J. Integr. Care.

[44] Zhang X., Han X., Dang Y., Meng F., Guo X., Lin J. (2017). User acceptance of mobile health services from users’ perspectives: The role of self-efficacy and response-efficacy in technology acceptance. Inform. Health Soc. Care.

[45] Lee H.Y., Lee Y.K., Kwon D. (2005). The intention to use computerized reservation systems: The moderating effects of organizational support and supplier incentive. J. Bus. Res..

[46] Zhao F., Ahmed F., Iqbal M.K., Mughal M.F., Qin Y.J., Faraz N.A., Hunt V.J. (2020). Shaping Behaviors Through Institutional Support in British Higher Educational Institutions: Focusing on Employees for Sustainable Technological Change. Front. Psychol..

[47] Sawrikar V., Mote K. (2022). Technology acceptance and trust: Overlooked considerations in young people’s use of digital mental health interventions. Health Policy Technol..

[48] Kim S.D. (2024). Application and challenges of the technology acceptance model in elderly healthcare: Insights from ChatGPT. Technologies.

[49] Kim E., Han S. (2021). Determinants of Continuance Intention to Use Health Apps among Users over 60: A Test of Social Cognitive Model. Int. J. Environ. Res. Public Health.

[50] Wu D., Gu H., Gu S., You H. (2021). Individual motivation and social influence: A study of telemedicine adoption in China based on social cognitive theory. Health Policy Technol..

[51] Cobb S., Dillard A., Yaghmaei E., Bazargan M., Assari S. (2025). Telehealth Perceived Benefits and Self-Efficacy Do Not Mediate the Effects of Demographic, Health, and Social Determinants on Telehealth Use of Low-Income African American and Latino Residents of Public Housing in Los Angeles. Healthcare.

[52] Hsia T.L., Chiang A.J., Wu J.H., Teng N.N., Rubin A.D. (2019). What drives E-Health usage? Integrated institutional forces and top management perspectives. Comput. Hum. Behav..

[53] Gabbiadini A., Paganin G., Simbula S. (2023). Teaching after the pandemic: The role of technostress and organizational support on intentions to adopt remote teaching technologies. Acta Psychol..

[54] von Elm E., Altman D.G., Egger M., Pocock S.J., Gøtzsche P.C., Vandenbroucke J.P. (2007). The strengthening the reporting of observational studies in epidemiology (STROBE) statement: Guidelines for reporting observational studies. PLoS Med..

[55] Njoku A., Mouloudj K., Bouarar A.C., Evans M.A., Asanza D.M., Mouloudj S., Bouarar A. (2024). Intentions to Create Green Start-Ups for Collection of Unwanted Drugs: An Empirical Study. Sustainability.

[56] Hair J.F., Hult G.T.M., Ringle C., Sarstedt M. (2017). A Primer on Partial Least Squares Structural Equation Modeling (PLS-SEM).

[57] Kamal S.A., Shafiq M., Kakria P. (2020). Investigating acceptance of telemedicine services through an extended technology acceptance model (TAM). Technol. Soc..

[58] Almokdad E., Mouloudj K., Njoku A., Bouarar A.C., Evans M.A., Asanza D.M., Mouloudj S. (2026). Exploring healthcare workers’ intentions to volunteer in digital healthcare services for underserved communities: An extended TAM-based study. Electron J. Gen. Med..

[59] Sriram S., Mohandoss H., Sunder N.P., Amirthalingam B. (2025). An Analysis of switching behavior from traditional hospital visit to E-health consultation. Healthcare.

[60] Gao D., Xu A., Yang L. (2025). Virtual care perceptions and experiences of older adults during COVID-19 in Canada: A Systematic Review. Healthcare.

[61] Wilson J., Heinsch M., Betts D., Booth D., Kay-Lambkin F. (2021). Barriers and facilitators to the use of e-health by older adults: A scoping review. BMC Public Health.

[62] Napetschnig A., Deiters W., Brixius K., Bertram M., Vogel C. (2025). Development and Evaluation of an Immersive Virtual Reality Application for Road Crossing Training in Older Adults. Geriatrics.

[63] Lam M.M.F., Wong F.M.F. (2026). Knowledge, attitudes, and practices of oral care for residential older people among healthcare providers in Macao. Geriatrics.

