# Supplementary material for: Determinants of Healthcare Professionals’ Intentions to Use Telemedicine for Elderly Care in Algeria: A Technology Acceptance Model Approach
Source: Geriatrics (Basel). 2026 Jun 27;11(4):76. doi: 10.3390/geriatrics11040076 (PMC13398288; doi:10.3390/geriatrics11040076)
Supplement: Supplementary file 1 [file geriatrics-11-00076-s001.zip › geriatrics-4308711-supplementary.pdf]

Manuscript Title: Determinants of Healthcare Professionals' Intentions to Use Telemedicine for Elderly Care in Algeria: A Technology Acceptance Model Approach

Study Design: Cross-sectional

Authors: Kamel Mouloudj, Anuli Njoku, Ahmed Chemseddine Bouarar, Dachel Martínez Asanza, Marian A. Evans and Snehal Baviskar

Journal: Geriatrics

Supplementary Table S1. STROBE Statement completed checklist

Section/Topic	Item No.	STROBE Recommendation	Location in Manuscript
**Title and abstract**	1	(a) Indicate the study's design with a commonly used term in the title or the abstract	(a) See title and Methods section first sentence (cross-sectional design)
		(b) Provide in the abstract an informative and balanced summary of what was done and what was found	(b) Abstract (includes design, methods, results, conclusions)
**Introduction**			
Background/rationale	2	Explain the scientific background and rationale for the investigation being reported	Section 1 (Introduction)
Objectives	3	State specific objectives, including any prespecified hypotheses	End of Introduction (primary and secondary objectives + research questions)
**Methods**			
Study design	4	Present key elements of study design early in the paper	Methods section opening: "cross-sectional survey design"
Setting	5	Describe the setting, locations, and relevant dates, including periods of recruitment, exposure, follow-up, and data collection	Methods → Sample and Procedure (Algeria; public or private hospitals; data collection period)
Participants	6	(a) Give the eligibility criteria, and the sources and methods of selection of participants. Describe methods of follow-up	(a) Methods → Sample and Procedure (inclusion criteria, exclusion criteria, recruitment)
		(b) For matched studies, give matching criteria and number of exposed and unexposed	(b) Not applicable
Variables	7	Clearly define all outcomes, exposures, predictors, potential confounders, and effect modifiers. Give diagnostic criteria, if applicable	Methods → Measures subsection (PU, PEOU, SE, IS, BI)
Data sources/ measurement	8	For each variable of interest, give sources of data and details of methods of assessment (measurement). Describe comparability of assessment methods if there is more than one group	Methods → Measures (Likert scales, item descriptions, validity, reliability)

Bias	9	Describe any efforts to address potential sources of bias	Methods → Notes on anonymity, self-administered format, and exclusion of incomplete surveys
Study size	10	Explain how the study size was arrived at	Methods → Sample and Procedure (rule-of-thumb for regression; n=130 sufficient)
Quantitative variables	11	Explain how quantitative variables were handled in the analyses. If applicable, describe which groupings were chosen and why	Methods → Data Analysis (all variables treated as continuous; regression approach)
Statistical methods	12	(a) Describe all statistical methods, including those used to control for confounding	(a) Methods → Data Analysis (descriptive statistics; skewness and kurtosis; VIF; hierarchical regression)
		(b) Describe any methods used to examine subgroups and interactions	(b) Not applicable (no subgroup analyses)
		(c) Explain how missing data were addressed	(c) Methods → Sample and Procedure (manual screening; exclusion of incomplete surveys; complete-case analysis)
		(d) If applicable, explain how loss to follow-up was addressed	(d) Not applicable (convenience sampling)
		(e) Describe any sensitivity analyses	(e) Not applicable
**Results**			
Participants	13	(a) Report numbers of individuals at each stage of study— e.g. numbers potentially eligible, examined for eligibility, confirmed eligible, included in the study, completing follow-up, and analysed	(a) Results → Description + Demographic characteristics (Table 2)
		(b) Give reasons for non-participation at each stage	(b) Not applicable
		(c) Consider use of flow diagram	(c) Not applicable. Use of a flow diagram was not deemed appropriate
Descriptive data	14	(a) Give characteristics of study participants (e.g. demographic, clinical, social) and information on exposures and potential confounders	(a) Results → Table of demographic characteristics (Table 2)
		(b) Indicate number of participants with missing data for each variable of interest	(b) None; complete-case analysis; stated in Methods
		(c) Summarise follow-up time (e.g. average and total amount)	(c) Not applicable
Outcome data	15	Report numbers of outcome events or summary measures over time	Results → Regression tables; descriptive statistics
Main results	16	(a) Give unadjusted estimates and, if applicable,	Results → Hierarchical

		confounder-adjusted estimates and their precision (e.g. 95% confidence interval). Make clear which confounders were adjusted for and why they were included	regression results
		(b) Report category boundaries if categorized variables were used	Not applicable (continuous variables)
		(c) If relevant, consider translating estimates of relative risk into absolute risk for a meaningful time period	(c) Not applicable (behavioral intention outcome)
Other analyses	17	Report other analyses done e.g. analyses of subgroups and interactions, and sensitivity analyses	Not applicable
**Discussion**			
Key results	18	Summarize key results with reference to study objectives.	Section 5.1 Discussion → first paragraph
Limitations	19	Discuss limitations and potential bias.	Section 5 (5.3. Limitations and Future Research)
Interpretation	20	Provide a cautious overall interpretation.	Section 5.1 Discussion + Section 6 Conclusions
Generalisability	21	Discuss generalizability (external validity)	Section 5 (5.3. Limitations and Future Research)
**Other Information**			
Funding	22	Give the source of funding and the role of the funders for the present study and, if applicable, for the original study on which the present article is based	This research received no external funding.
